# Cutaneous Melanoma in Adolescents and Young Adults Versus Older Patients: Clinical and Histopathological Differences in Western Romania

**DOI:** 10.3390/dermatopathology13020027

**Published:** 2026-06-20

**Authors:** Bianca Roxana Natarâş, Sorina Maria Tăban, Aura Jurescu, Octavia Cornelia Viţa, Remus Florin Cornea, Ioana Hurmuz, Adelina Vidac, Daciana Grujic, Valentin Tudor Popa, Alis Liliana Carmen Dema

**Affiliations:** 1 Anapatmol Research Centre, Victor Babeş University of Medicine and Pharmacy, 300041 Timişoara, Romania; 2 Doctoral School, “Victor Babeş” University of Medicine and Pharmacy, Eftimie Murgu Square No. 2, 300041 Timişoara, Romania; 3 Department of Plastic Surgery, Victor Babeş University of Medicine and Pharmacy, 300041 Timişoara, Romania

**Keywords:** melanoma, AYA, young adults

## Abstract

Aim: This study aimed to identify the clinical and pathological features of primary cutaneous melanomas in young patients, comparing them with those of older patients. Materials and Methods: We performed a retrospective study observing the differences with respect to clinical and pathological features in young patients versus older patients. We distributed the cases into two groups: patients < 40 years diagnosed with cutaneous melanoma and patients ≥ 40 years diagnosed with cutaneous melanoma. Results: From the total number of primary cutaneous melanomas diagnosed, 11% of cases were represented by young patients. The clinical and pathological features more frequently associated with cutaneous melanomas in AYAs (adolescents and young adults) were represented by the superficial spreading subtype (*p* = 0.0003), a brisk inflammatory infiltrate (*p* = 0.0061), a pT1–pT2 pathological stage (*p* = 0.0183), decreased mitotic activity (*p* = 0.0186), decreased Breslow index (*p* = 0.0301), and female sex (*p* = 0.022). Conclusions: The most important features of cutaneous melanomas diagnosed in AYA patients were represented by the superficial spreading subtype, the presence of a brisk inflammatory infiltrate, and a pT1–pT2 pathological stage.

## Simple Summary

This study compared clinical and pathological features of primary cutaneous melanoma in younger patients (under 40 years) versus older patients. In our study, 11% of the cases affected younger patients. In this population, melanomas were more often of the superficial spreading histological subtype, presented brisk inflammatory infiltrate more often, were diagnosed more frequently in earlier stages (pT1–pT2), and showed a lower mitotic count and a lower Breslow index. Younger patients tended to have less aggressive melanoma features compared with older patients.

## 1. Introduction

Malignancy in adolescents and young adults (AYAs) is defined by the National Cancer Institute as a diagnosis that affects patients aged 15 to 39 years [1]. Cutaneous melanoma (CM) is rarely diagnosed in young people, with an incidence of 1/1,000,000 in patients ≤ 14 years, increasing to 71/1,000,000 in adolescents and young adults [2]. With an annual incidence rate of 20 per 100,000, melanoma currently represents one of the most frequent malignant tumors in adults, and the second most frequent in patients aged 0 to 39 years old, in both sexes [3,4]. Melanomas represent 5.3% of all cancers diagnosed in patients from 10 to 19 years and 12.4% of all malignancies diagnosed in patients between 20 and 29 years old [5,6,7]. In young adults (adults under 40 years old), melanoma is accountable for more than 11% of the diagnosed malignancies, representing the second most frequently diagnosed malignant tumor, after breast cancer [8,9]. In developed countries, the melanoma incidence has increased more rapidly than any other malignancy in the last 50 years [10,11,12,13,14]. One of the most important contributors to the increasing incidence of melanoma in younger populations is the increased use of indoor tanning devices. Tanning beds use before the age of 30 has been linked with a 75% elevated risk of developing melanoma. This behavior, associated with tanned skin being perceived as a beauty standard, contribute to an increasing incidence in this age category [15]. Young adults, especially white female adolescents from higher socioeconomic backgrounds, present the highest rate of indoor tanning use—an additional source of UV radiation [16].

There are four main protection strategies: avoiding indoor tanning; avoiding exaggerated outdoor activities between 10:00 AM and 4:00 PM; using protective clothing such as hats, sunglasses, long pants, and long-sleeved shirts; and daily use of sunscreen with a sun protection factor (SPF) greater than 15 [17].

Multiple personal factors that increase the risk of developing melanoma are well known, including blue eyes, red hair, fair skin, multiple moles, and family history of freckles [18].

Studies show that age independently predicts disease-free (the time from treatment initiation to cancer recurrence or death) and overall survival (the time a patient stays alive from diagnosis or treatment) [19,20]. Young adults have a 5-year survival rate that is 94.6% higher than the overall population [21].

Young patients survive more often because their melanomas are usually thin and localized. Melanomas with regional spread or metastases have a poor prognosis in all age groups [22].

This study compared the clinical and morphological features of cutaneous melanomas in adolescents and young adults (AYAs) with those in older patients.

## 2. Materials and Methods

We obtained the ethics approval from the ethics committee of “Victor Babes” University of Medicine and Pharmacy Timișoara, approval number: 90, date: 19 December 2022. It was mandatory to obtain the ethics approval before the beginning of the study (19 December 2022). Study data was retrospectively reviewed up to 2024.

We obtained informed consent in accordance with Annex 4 of the Norms for the application of Law 104/2003 in Romania. The patients provided a written agreement upon admission to the hospital to use tissue and images of the tissue for scientific purposes and for publication in medical studies, provided that identifiable patient information is not disclosed.

We performed a retrospective observational study, identifying all the cases with a primary cutaneous melanoma histopathological diagnosis, from January 2018 to December 2023, from the databases of two institutions in Western Romania.

Inclusion criteria: invasive primary cutaneous melanomas with a complete histopathological report.

Exclusion criteria: invasive primary cutaneous melanomas without a complete histopathological report, in situ melanomas.

After the case selection, we introduced the clinical and pathological parameters of the primary cutaneous melanomas in an Excel table.

We divided the cases into two groups:

- Primary cutaneous melanomas diagnosed in patients < 40 years (the first group/AYAs group);
- Primary cutaneous melanomas diagnosed in patients ≥ 40 years (the second group).

We analyzed and compared the following clinical and pathological features between the two groups: age, sex, primary tumor location, histological subtype, pathological stage, Breslow index, Clark level, mitotic rate, ulceration and regression status, inflammatory infiltrate type (brisk/non-brisk), perineural invasion, lymphovascular invasion, the presence/absence of satellites and in transit metastases, lymph node status, distant metastases, and associated nevus.

The vertical growth phase in melanoma is defined by the presence of cohesive collections of melanoma cells in the dermis, which are often larger or cytologically distinct from overlying cells, indicating a significant, higher-risk phase of progression.

The mitotic rate was recorded in a “hot spot,” which is the area with the highest density of mitotic activity, counting the total number of mitoses per square millimeter.

We considered histological regression in melanoma as the partial or complete disappearance of melanoma cells from the dermis, significant dermal fibrosis, increased small ectatic blood vessels, dense lymphohistiocytic infiltrate, presence of melanophages, and attenuated epidermis.

We considered a brisk inflammatory infiltrate when the lymphocytes actively infiltrated the entire base of the tumor or when the lymphocytes were distributed diffusely throughout the entire tumor mass. A non-brisk inflammatory infiltrate was assigned when the lymphocytes were only present in scattered, focal groups rather than covering the entire base or mass of the tumor.

Sentinel lymph node biopsy (SLNB) in young patients is routinely offered for all pT2 melanomas and select pT1b melanomas, while for pT1a tumors, its use is guided by high-risk features and the patient’s young age. The procedure is considered in younger patients if specific adverse, high-risk histological features are present (≥2 mitoses/mm^2^, lymphovascular invasion, positive or insufficient deep margins). Not all the cases in our study presented those features. The SNLB represented the surgeon’s decision.

The excised specimens were placed in formalin, embedded in paraffin, and stained with hematoxylin–eosin stain in the pathology departments.

IHC Analysis

For some of the cases, immunohistochemical (IHC) markers were used for the diagnosis. For the IHC analysis, additional sections from the selected paraffin blocks, with a thickness of 3–4 µm, were placed on Super Frost Ultra Plus slides. We used the following primary antibodies: S100 [polyclonal—DAKO, RTU], Melan A (clone A103—DAKO, ready to use (RTU), HMB45 (clone HMB45—DAKO, RTU), and Sox 10 (clone EP268—Dako, RTU). Antigen retrieval was performed by heat-induced epitope retrieval (HIER) in target retrieval solution pH6 (for S100 and Sox 10), pH9 (for Melan A), and enzymatic pretreatment for HMB45 for 20 min at 98 °C.

The statistical analysis of the evaluated parameters was performed using the functions in Microsoft Excel 2010 and GraphPad Prism software, version 9.5.1. To analyze differences across parameters, we used Fisher’s exact test and the chi-square test. The results were considered statistically significant when the *p* value was <0.05. Due to the relatively small number of young patients (*n* = 22) and the low prevalence of some clinicopathological characteristics, Fisher’s exact test was used for categorical comparisons when the assumptions of the chi-square test were not met. This approach ensured valid estimation of statistical significance despite small expected frequencies in contingency table cells. Fisher’s exact test was applied when expected cell frequencies were less than 5, particularly for infrequent clinical and histopathological features in the younger melanoma cohort.

## 3. Results

The clinical and pathological features of the cases are presented in Table 1.

From the total number of cases, the young patients diagnosed with cutaneous melanomas represented a minority of cases—11% (22/204)—while the older patients represented 89% of cases (182/204). In the first group, 77% (17/22) of the patients were represented by females and 23% (5/22) by males (female: male ratio—3.4:1). In the second group, the female and male patients represented 50% each (female: male ratio—1:1). We obtained a statistically significant *p* value (*p* = 0.022).

**Figure 1:**
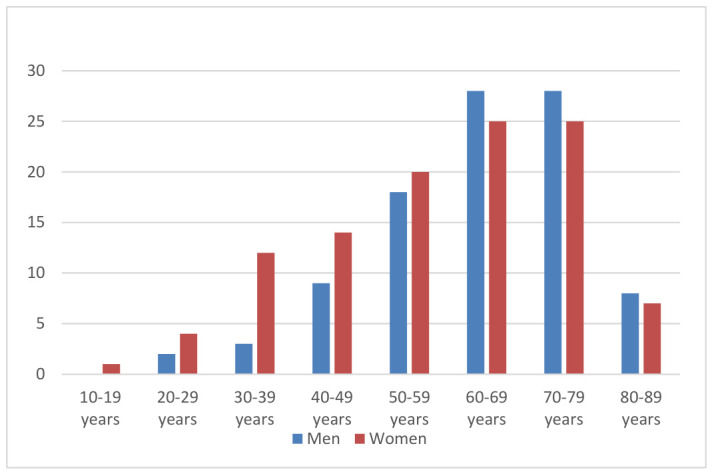
The distribution of the cases based on sex and age.

In the first group, the mean age was 31.27 years (age range: 17–39 years). In the second group, the mean age was 64.54 years (age range 40–86 years). In the first group, the majority of the patients were aged between 30 and 39 years—68% of patients (15/22). In the second group, the majority of the patients were diagnosed in the age intervals of 60–69 years and 70–79 years, each composed of 29% of patients (53/182 each)—see Figure 1.

In the first group, the cutaneous melanomas were located more often on the lower extremities—36% of patients (8/22)—followed by the ones located on the trunk and the head and neck area—23% each (5/22 each)—and the upper extremities—18% (4/22). In the second group, the main location was represented by the trunk—42% of patients (77/182)—followed by the lower extremities—22% (39/182)—and the head and neck area and the upper extremities—18% each (33/182 each). The *p* value was not statistically significant (*p* = 0.262).

The main anatomical location in the female patients from the first group was represented by the lower extremities—35% of patients (6/17)—followed by the trunk area—30% (5/17)—and the head and neck and upper extremities—17.5% each (3/17 each). The male patients of the same group presented a predilection for the head and neck area and the lower extremities—40% of patients in each area (2/5 each)—with 20% of the melanomas developing on the upper extremities (1/5). The *p* value was not statistically significant (*p* = 0.5020)—see Table 2.

The main anatomical location in the female patients from the second group was represented by the trunk—35% of patients (32/91)—followed by the lower extremities—29% (26/91)—the upper extremities—21% (19/91)—and the head and neck area—15% (14/91). The male patients of the same group also presented a predilection for the trunk area—49% of patients (45/91). The next locations, in order of frequency, were represented by the head and neck area—21% of patients (19/91)—the upper extremities—15% (14/91)—and the lower extremities—14% (13/91). The *p* value was statistically significant (*p* = 0.0461)—Table 3.

The superficial spreading melanoma in vertical growth phase was the main histological subtype in both groups: in the first group, it represented 59% of the cases (13/22) and 55% of cases in the second group (100/182). The nodular subtype was rarely diagnosed in the first group—14% of cases (3/22)—while the second group was diagnosed more often—32% (58/182). The superficial spreading melanomas were more frequently diagnosed in the first group—27% of cases (6/22), compared with the second group—4% (8/182). The acral subtype was absent in the first group, representing 8% of the second group’s cases (16/182). We obtained a statistically significant *p* value for this parameter—*p* = 0.0003.

Melanomas with a Breslow index < 1 mm represented a higher percentage in the first group—45% of patients (10/22)—compared with the second group—19% (53/182). Melanomas with a Breslow index > 4 mm were rarely diagnosed in the first group—23% of patients (5/22)—compared with the second group—44.5% (81/182). The *p* value was statistically significant—*p* = 0.0301. The median Breslow index in the first group was significantly lower—2.18 mm, compared with the second group—4.6 mm.

In the first group, half of the cases presented a Clark level II or III, while the other half presented a Clark level of IV or V—50% each. In the second group, the cases with a Clark level of IV or V predominated—74.5% of cases (135/182). The *p* value was statistically significant (*p* = 0.0242).

In the first group, the majority of the cases were pathologically staged pT1 or pT2—64% of cases (14/22), while in the second group, the advanced stage melanomas predominated (pT3 and pT4)—65% (118/182). The *p* value was statistically significant (*p* = 0.0183).

The percentage of the cases that presented lymphovascular invasion was lower in the first group—4.5% of cases (1/22)—compared with the second one—7.7% (14/182). The *p* value was not statistically significant (*p* > 0.999). In the first group, 4.5% of the cases presented perineural invasion (1/22), while in the second group, the percentage was higher—9.3% (17/182). The *p* value was not statistically significant (*p* = 0.6996).

Only 23% of the cases in the first group (5/22) presented a mitotic activity > 4 mitoses/mm^2^ compared with the second group, where a > 4 mitoses/mm^2^ mitotic rate predominated—50% (91/182). The *p* value was statistically significant with *p* = 0.0186.

In the first group, the majority of the cases presented a brisk inflammatory infiltrate—59% of cases (13/22)—compared with the second group, which contained a minority of melanomas with this type of inflammatory infiltrate—28% (51/182). The *p* value was statistically significant (*p* = 0.0061). The assessment of the inflammatory infiltrate was purely morphological.

In the first group, 50% (11/22) of the melanomas were ulcerated, a smaller percentage compared with the second group—61% (111/182). The *p* value was not statistically significant (*p* = 0.3614).

In the first group, regressive changes were present in 36% of the cases (8/22), while in the second group, 42% of the cases (77/182) presented those aspects. The *p* value was not statistically significant (*p* = 0.652).

In the first group, 36% (8/22) of the melanomas were associated with melanocytic nevi, while in the second group, the percentage was lower—19% (35/182). The *p* value was not statistically significant (*p* = 0.0924).

In the first group, in 73% of the cases (16/22), a sentinel lymph node excision was performed—Table 4—compared with 56% of the cases (102/182) in the second group. The *p* value was not statistically significant (*p* = 0.1719). A lower percentage of the first group patients presented lymph node metastases—45% of patients (5/11)—compared with the second group—59% (38/64). The *p* value was not statistically significant (*p* = 0.5128).

In the first group, 9% (2/22) of the cases presented microsatellites; in the second group, a higher percentage of the cases—24% (44/182)—presented microsatellites, satellites, and/or in-transit metastases. The *p* value was not statistically significant (*p* = 0.1741).

In the AYA group, 5% (1/22) of the patients presented distant metastasis (cerebral metastasis). In the second group, 3% of the patients (6/182) presented distant metastases: brain metastases (*n* = 2), small bowel metastases (*n* = 2), adrenal gland metastasis (*n* = 1), and distant cutaneous, subcutaneous, and breast metastases (*n* = 1). The *p* was not statistically significant (*p* = 0.5558).

## 4. Discussion

The incidence of cutaneous melanomas in AYA patients was 9.9% in a study [23]. We obtained similar results in the same population in our study with 11% incidence. In another study, the incidence of melanoma in women was twice compared with men in young patients [24]. In our study, in the young patients’ group, we observed an increased incidence of melanoma in women compared with men (3.4:1), a result different from other studies. This may be in part attributed to the increased use of indoor tanning among females, which is linked with an increased melanoma risk [25,26,27]. In other studies, the melanoma incidence in men was greater than in women after 40 years [28,29,30,31]. We observed the same melanoma incidence in men and women in patients ≥ 40 years.

In our study, the lower extremities were the main site of melanoma development in AYA patients, the trunk being affected more often in the older population. Some studies obtained different results regarding the anatomic location of the melanoma, in which young patients developed melanomas more frequently in the trunk—an intermittently sun-exposed area linked with sporadic intense UV exposure (tanning)—while older adults presented melanomas more frequently in the head and neck area—a chronically sun-exposed region, caused by a lifetime of cumulative sun damage [32,33].

In other studies, melanomas developed more frequently in the trunk region in young patients for both females and males [28]. Those results are different compared with our study, where the main anatomical location in the female patients from the first group was represented by the inferior extremities, while the male patients of the same group presented a predilection for the head and neck area and the inferior extremities.

Similar to other studies [34], the superficial spreading melanoma subtype was diagnosed more often in the first group and acral and nodular melanomas in the second group. The mean Breslow index in our study in patients < 40 years was increased (2.18 mm) compared with other studies (−1.05 mm) [35]. The higher Breslow index mean in our study can be attributable to the lack of screening programs for melanoma and the low level of medical education in the general population.

In our study, the percentage of Clark level II and III was lower (50%) in the first group compared with other studies, which registered a Clark level of II or III in 82% of the cases of the AYA patients [36]. Other studies obtained a higher percentage of AYA patients with pT1 and pT2 pathological stages (85%) compared with our study (64%) [34]. This implies a deeper invasion in a higher percentage of our cases. In other studies, the percentage of lymphovascular invasion was higher (8.8%) compared with our study (4.5%) [37].

The TILs activation and their ability to destroy malignant cells or liberate immune-activating molecules contribute to the control and suppression of the tumor development [38,39]. The brisk inflammatory infiltrate was present in the majority of the cutaneous melanomas in young patients and only in a minority of the older population in our study, implying an increased immunogenic response in the young population.

In our study, the percentage of ulcerated melanomas in AYA patients was significantly higher (50%) compared with other studies (12.8%) [23]. Regression observed in primary cutaneous melanoma represents an immunological event that leads to partial or total disappearance of the tumor [40]. Histologic regression is present in 10% to 35% of all primary cutaneous melanomas [41]. In our AYAs population, the percentage of melanomas with regression was double (36%) compared with other studies (18.4%) [23].

Although the majority of the melanomas arise de novo, a notable subset is associated with pre-existing melanocytic nevi, an occurrence that has significant implications in the understanding of melanoma development and clinical practice [42,43,44,45]. In our study, a higher percentage of young patients were diagnosed with nevus-associated melanomas compared with the older population. Our results are similar with those obtained in other studies, which affirm that nevus-associated melanomas are diagnosed more frequently in younger patients and often develop on the trunk [43,46].

In the primary cutaneous melanoma, the main risk factors of sentinel lymph node (SLN) metastasis are Breslow thickness, ulceration, and mitotic rate. If a patient has a risk of a positive SLN of less than 5% (e.g., MIS, T1a, non-ulcerated lesions with a Breslow index less than 0.8), the NCCN does not recommend sentinel lymph node biopsy (SLNB). For patients with a 5–10% risk (T1b melanoma, 0.8–1.0 mm Breslow thickness, or less than 0.8 mm with ulceration), the NCCN recommends discussing SLNB. When the probability of a positive SLN is greater than 10% (T2a–T4b melanoma, Breslow index greater than 1.0 mm), the NCCN advises both discussing and performing SLNB [47]. Recurrence and death have a higher probability to arise in sentinel node-positive cases of AYA patients, similar with the cases of older adults [48,49].

Our study presented different results compared with other studies regarding the lymph node metastases incidence. In our study, the incidence was significantly increased in young patients—45% of patients—compared with other studies (8%). This also happened for the older population. In our study, the percentage of cases that involved lymph nodes was 59% compared with other studies (21%) [50].

Metastatic melanoma is a lethal disease with a rapid systemic spreading. The 5-year survival rate is less than 15% in patients with metastatic dissemination [51]. Patients with only one distant metastatic site involvement have a considerably improved outcome in comparison with patients with at least two distant sites [52].

### Study Limitations

One limitation is represented by the lack of complete information about the oncological treatment and survival. Another limitation is represented by the small number of young patients diagnosed with cutaneous melanomas. The adolescent population is not well represented in this study because the majority of the patients from this age group are evaluated in another hospital.

The univariate analyses are limiting the validity of the conclusions.

## 5. Conclusions

The most important features of cutaneous melanomas diagnosed in AYA patients were represented by the superficial spreading subtype, the presence of a brisk inflammatory infiltrate, and a pT1–pT2 pathological stage.

## Figures and Tables

**Table 1:** The clinical and pathological features of primary cutaneous melanomas.

		<40 Years		≥40 Years		
		N = 22	%	N = 182	%	
Sex	Female	17	77	91	50	
	Male	5	23	91	50	*p* = 0.022
Anatomic location	Head and neck	5	23	33	18	
	Upper extremities	4	18	33	18	
	Lower extremities	8	36	39	22	
	Trunk	5	23	77	42	*p* = 0.262
Histologic subtype	Superficial spreading	6	27	8	4	
	Superficial spreading In vertical growth phase	13	59	100	55	
	Nodular	3	14	58	32	
	Acral	0	0	16	8	*p* = 0.0003
Breslow index	<1 mm	10	46	35	19	
	1–2 mm	4	18	28	15	
	2.01–4 mm	3	14	38	21	
	>4 mm	5	23	81	45	*p* = 0.0301
Clark level	II–III	11	50	47	25.5	
	IV–V	11	50	135	74.5	*p* = 0.0242
pT	pT1–pT2	14	64	64	35	
	pT3–pT4	8	36	118	65	*p* = 0.0183
Lymphovascular invasion	Absent	21	95.5	168	92.3	
	Present	1	4.5	14	7.7	*p* > 0.999
Perineural invasion	Absent	21	95.5	165	92.7	
	Present	1	4.5	17	9.3	*p* = 0.6996
Mitotic rate	0–1/mm^2^	7	32	24	13	
	2–4/mm^2^	10	45	67	37	
	>4/mm^2^	5	23	91	50	*p* = 0.0186
Tils	Brisk	13	59	51	28	
	Non-brisk	9	41	131	72	*p* = 0.0061
Ulceration	Absent	11	50	71	39	
	Present	11	50	111	61	*p* = 0.3614
Regression	Absent	14	64	104	57	
	Present	8	36	77	43	*p* = 0.652
Satellites	Absent	20	90	138	76	
	Present	2	10	44	24	*p* = 0.1741
Associated nevus	Absent	14	64	147	81	
	Present	8	36	35	19	*p* = 0.0924
Distant metastases	Absent	21	95	176	97	
	Present	1	5	6	3	*p* = 0.5558
Lymph node excision	Present	16	73	102	56	
	Absent	6	27	80	44	*p* = 0.1719
		**N = 16**	**%**	**N = 106**	**%**	
Lymph node metastases	Absent	11	67	64	60	
	Present	5	33	38	40	*p* = 0.5128

**Table 2:** Anatomical location of cutaneous melanomas in patients < 40 years.

Anatomical Location	Men	Women	
Head and neck	2	3	
Upper extremities	1	3	
Lower extremities	2	6	
Trunk	0	5	*p* = 0.5020

**Table 3:** Anatomical location of cutaneous melanomas in patients ≥ 40 years.

Anatomical Location	Men	Women	
Head and neck	19	14	
Upper extremities	14	19	
Lower extremities	13	26	
Trunk	45	32	*p* = 0.0451

**Table 4:** Clinicopathological features the melanomas in the AYA group with sentinel lymph node excision.

Sex	Age	Tumor Location	Histological Subtype	pT	pN	Lymphovascular Invasion	Perineural Invasion	Breslow Index (mmm)	Clark Level	Ulceration	Regression	Inflammatory Infiltrate Type	Mitotic Rate/1 mm^2^
F	33	Lower extremities	Superficial spreading	pT2a	N0	absent	absent	1.8	IV	absent	absent	Brisk	4
F	37	Lower extremities	Superficial spreading in vertical growth phase	pT2b	N0	absent	absent	1.6	III	present	present	Non-brisk	2
M	28	Head and neck	Superficial spreading in vertical growth phase	pT2a	N0	absent	absent	1.2	III	absent	absent	Brisk	4
F	30	Upper extremities	Nodular	pT4b	N2b	absent	absent	5.5	IV	present	present	Brisk	10
F	34	Trunk	Superficial spreading	pT1a	N0	absent	absent	0.65	III	absent	present	Brisk	0
F	32	Trunk	Superficial spreading	pT1a	N0	absent	absent	0.4	III	absent	absent	Brisk	0
F	17	Lower extremities	Superficial spreading in vertical growth phase	pT4b	N1	absent	absent	4.8	IV	present	absent	Non-brisk	2
M	35	Upper extremities	Superficial spreading in vertical growth phase	pT1b	N0	absent	absent	0.6	III	present	absent	Brisk	0
F	34	Lower extremities	Superficial spreading in vertical growth phase	pT3b	N1a	absent	absent	2.5	IV	present	absent	Non-brisk	2
M	39	Lower extremities	Superficial spreading in vertical growth phase	pT1b	N0	absent	absent	0.9	III	absent	absent	Brisk	1
F	39	Upper extremities	Superficial spreading	pT1a	N0	absent	absent	0.5	II	absent	present	Brisk	0
F	31	Head and neck	Superficial spreading in vertical growth phase	pT4b	N0	absent	absent	5.4	IV	present	present	Non-brisk	3
F	29	Head and neck	Superficial spreading	pT1a	N0	absent	absent	0.8	IV	absent	absent	Non-brisk	0
M	28	Lower extremities	Superficial spreading In vertical growth phase	pT1b	N0	absent	absent	0.6	III	present	absent	Brisk	2
F	30	Trunk	Nodular	pT4b	N1a	absent	absent	5.2	IV	present	absent	Non-Brisk	7
F	30	Lower extremities	Nodular	pT4b	N1a	absent	absent	6	IV	present	present	Non-brisk	9

## Data Availability

All data generated or analyzed during this study are included in this published article and can be provided if needed or requested by the reviewer.

## Abbreviations

The following abbreviations are used in this manuscript:

AYAs	Adolescents and Young adults
SNL	Sentinel lymph node
SNLB	Sentinel lymph node biopsy
NCCN	National comprehensive cancer network
